# Nonlinear Optimization of Light Field Point Cloud

**DOI:** 10.3390/s22030814

**Published:** 2022-01-21

**Authors:** Yuriy Anisimov, Jason Raphael Rambach, Didier Stricker

**Affiliations:** 1 Department of Augmented Vision, German Research Center for Artificial Intelligence, Trippstadter Str. 122, 67663 Kaiserslautern, Germany; Jason.Rambach@dfki.de (J.R.R.); Didier.Stricker@dfki.de (D.S.); 2 Department of Computer Science, Technical University of Kaiserslautern, 67663 Kaiserslautern, Germany

**Keywords:** light field, depth estimation, point cloud

## Abstract

The problem of accurate three-dimensional reconstruction is important for many research and industrial applications. Light field depth estimation utilizes many observations of the scene and hence can provide accurate reconstruction. We present a method, which enhances existing reconstruction algorithm with per-layer disparity filtering and consistency-based holes filling. Together with that we reformulate the reconstruction result to a form of point cloud from different light field viewpoints and propose a non-linear optimization of it. The capability of our method to reconstruct scenes with acceptable quality was verified by evaluation on a publicly available dataset.

## 1. Introduction

A definition of a light field was first given by Gershun in [1]. The light field is formed from all light rays, which are passing through all points in space in all directions. It was generalized in the work of Adelson and Bergen [2], where the sufficiency of finite light rays sampling was stated. For computer vision tasks, based on the two-plane parameterization from [3], light field can be considered as a one- or two-dimensional set of two-dimensional images, called light field views, and captured with the preservation of fixed physical distance between them.

Various devices can be used for light field acquisition. For static scenes, an ordinary perspective camera can be moved on certain distances for capturing a scene from multiple viewpoints. In cases when ensuring of equal camera movements is not possible, a control pattern can be used with two oppositely oriented cameras [4]. For dynamic scenes and varying resolutions, two different configurations can be used.

A light field camera can consist of multiple isolated camera sensors and lenses [5] or of one camera sensor and multi-lens arrays in front of it [6]. Such an approach evolves to the micro-lens array, where the light field images are captured as the small views from many viewpoints [7]. Modern configurations can be downscaled to the form-factor of mobile cameras [8].

Various features of light field images attract the attention for different research and industrial applications. For instance, digital refocusing allows changing the focus of the captured image [7]. In addition, light fields can be used as a source for the accurate novel view synthesis [9].

Additionally, the presence of multiple views in the light field can be used for the scene three-dimensional reconstruction. One important feature of the light field cameras for that purpose is related to the view alignment. Capturing units of light field camera are oriented in the same direction and the distance between them is known and fixed, which allows simplifying the search of matching correspondence among light field views.

**Figure 1:**
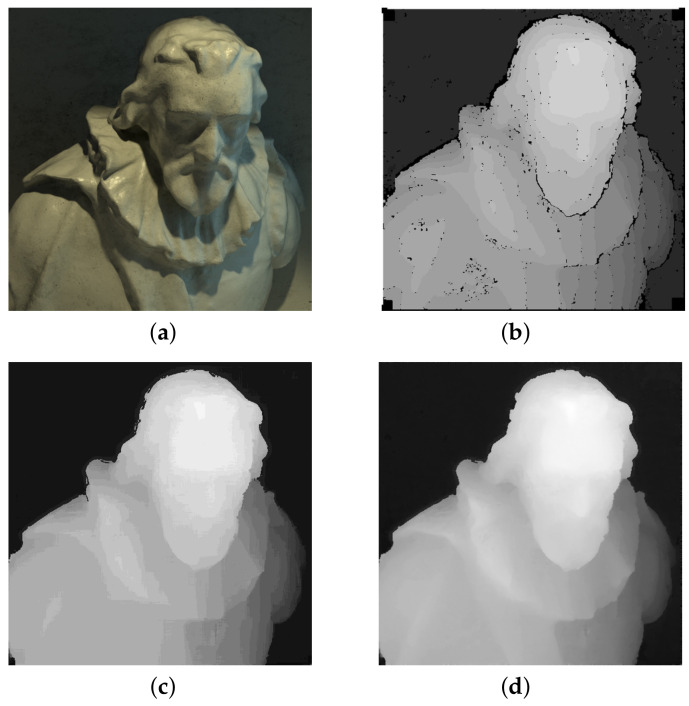
Results of our algorithm for a synthetic scene "cotton" from 4D Light Field Benchmark [11]: (**a**) center image from a light field, (**b**) initial disparity map, (**c**) final disparity map, (**d**) refined disparity map.

In this work, we present an extension of our depth estimation algorithm from [10]. An example of the result of our algorithm is demonstrated in Figure 1. The contributions compared to this algorithm are:

- per-layer disparity filtering and color-consistency based holes filling, which noticeably improves the accuracy of initial disparity map,
- different representation of the reconstruction result in the form of point cloud, with the additional step of its nonlinear refinement over light field views.

## 2. Related Work

Many methods exist for solving the light field depth estimation task. Wanner and Goldluecke in [12] propose the labeling of epipolar-plane images (EPIs) [13] with variational methods, introducing the structure tensor for its analysis. Neri et al. in [14] provides a combination of multi-resolution multi-view stereo and variational methods for solving depth estimation tasks. A method which takes pixel occlusions into consideration was proposed by Wang et al. in [15].

Johannsen et al. in [16] construct the light field dictionaries with specific disparity values based on EPIs. Strecke et al. [17] estimate depth and normals using partial focal stacks with their joint optimization. Preceding methods [10,18] used a construction of matching cost volume in the bordered space with further cost refinement.

In the method of Shin et al. [19], a multi-stream architecture of EPI analysis for depth estimation is presented. Huang et al. [20] proposed a model for disparity estimation based on multi-scale cost aggregation with additional edge guidance.

Few studies have been published on the point cloud utilization for light fields particularly. In their analysis, Perra et al. [21] show the object extraction and point cloud estimation from depth maps together with a comparison of point clouds, retrieved from the two popular plenoptic cameras. In the work of Ferreira et al. [22], the RANSAC method is applied to SIFT-based features from plenoptic images for estimating the virtual point cloud. This point cloud is back-projected to the micro-lenses space and further optimized using least squares.

An approach of Farhood et al. [23] shows how the depth map, obtained by the light field camera, can be improved for getting the high-quality point cloud. The depth map is modified by histogram manipulations, aimed for better separation of depth layers, and then further enhanced by adding information about the objects’ edges. This provides better separation of different objects in a point cloud. In the method of Yucer et al. [24], the point cloud is reconstructed from patch-based local light field gradient information.

Light field point cloud estimation for the case of unfocused light field can be considered as the extension of stereo to multi-view with strict baseline constraints. A classical work in this direction was published by Liu et al. [25]. The estimation process contains the detection of initial point clouds by stereo matching, their merging with downsampling and further mesh generation. The construction of experimental camera, used in this publication, can be considered as a combination of sparse focused light field cameras due to capturing devices placement.

In the last few years, many approaches have been utilizing deep learning methods for point cloud reconstruction. A noticeable publication in this direction was published by Chen et al. [26] and presents a two-stage method of multi-view point cloud estimation. First, the coarse depth map is estimated by using MVSNet [27]. It is used as a component for loss estimation and as an initial estimation for the point cloud. This point cloud is augmented based on image feature pyramid, extracted from the provided views; and iteratively refined based on the PointFlow network, proposed in that paper.

## 3. Initial Disparity Map Generation

This section describes the steps for getting an initial disparity map, which is used afterwards as an initialization step for the further nonlinear optimization. For that, we follow and improve the disparity estimation algorithm, described in [10].

### 3.1. Light Field Parameterization

A classical way of representing the light rays was defined by Adelson and Bergen in [2]. They were parameterizing rays by a plenoptic function, consisting of three dimensions for the ray position and two dimensions for its orientation. However, this description might not be very convenient for the utilization in computer vision algorithms due to its complexity.

**Figure 2:**
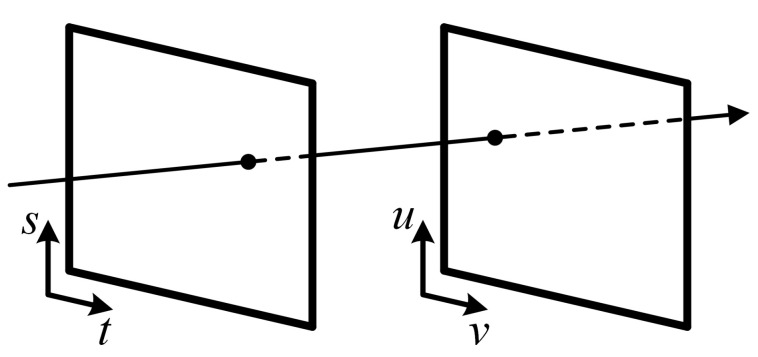
Two-plane light field parameterization from [3].

One of the common descriptions of light field exists in a form of two-plane parameterization, proposed by Levoy and Hanrahan in [3]. Following this definition, every ray of a light field is described by the intersection over a plane of spatial coordinates (u,v) and an angular coordinates plane (s,t) , as demonstrated in Figure 2. We denote the light field as *L*, with a specific ray projection as L(u,v,s,t) .

### 3.2. Matching between Light Field Views

In the context of computer vision, the light field is considered as a set of two-dimensional images assembled into a two-dimensional array. Individual rays of the light field are projected onto these images in the form of pixels. To recover the depth information associated with each light field ray, we use pixel similarity measurement techniques. It is convenient to use a concept of "disparity", which is inversely proportional to the depth, as disparity is expressed as a distance in pixel units and can be calculated explicitly from the light field views. Based on the two-plane parameterization, the matching position of a certain pixel $\left(\hat{u},\hat{v}\right)$ from a given reference light field view $\left(\hat{s},\hat{t}\right)$ with the disparity *d* can be found in arbitrary light field view (s,t) as [28]:

(1) $$p\left(u,v,s,t,d\right)=L(\hat{u}+\left(\hat{s}-s\right)d,\hat{v}+\left(\hat{t}-t\right)d,s,t).$$

### 3.3. Reference and Anchor Views

**Figure 3:**
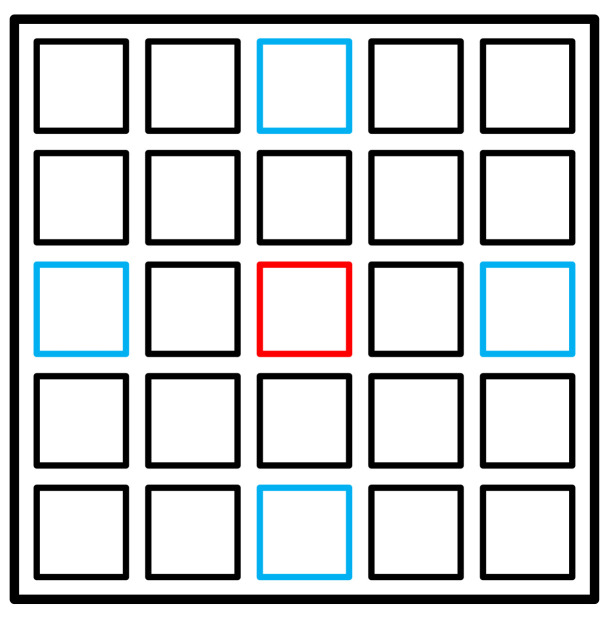
Reference (red) and anchor (blue) views of the light field.

Our disparity estimation approach starts with the computing of coarse disparity maps for "reference" view from the "anchor" views of the light field. For reference, we select a view, which lies in the middle of both light field angular axes. As anchors, we define the views at the borders of the light field, which are lying on the cross with the reference view in its center. Figure 3 illustrates how the reference and anchor views are placed in the light field image. These views are important for the disparity estimation as they cover all visible scene points, projected to the light field image.

To generate the set of coarse disparity maps, these views are utilized pairwise. Based on the reference light field view with coordinates $\left(\hat{s},\hat{t}\right)$ , we define a set of four cross-lying views in the light field image $V=\{\left(\hat{s},t_{min}\right),\left(\hat{s},t_{max}\right),\left(s_{min},\hat{t}\right),\left(s_{max},\hat{t}\right)\}$ , where $s_{min}$ , $t_{min}$ , $s_{max}$ , $t_{max}$ corresponds to minimal and maximal possible indexes on vertical (*s*) and horizontal (*t*) angular dimensions of the light field image.

### 3.4. Matching Cost Generation

A matching cost can be described as a three-dimensional structure, where every element represents the comparison of every pixel in reference view with the corresponding pixel in another view based on the disparity hypothesis, lying in the range of $T=[d_{min},d_{max}]$ , where $d_{min}$ and $d_{max}$ are the minimum and maximum disparity hypotheses.

Different methods exist to measure pixel similarity. Roughly, these methods can be divided into pixel-wise and window-wise. Commonly used pixel-wise functions are Manhattan and Euclidean distances [29]. Widely used window-wise measures are the sum of absolute differences, the sum of squared differences and normalized cross-correlation [30]. Window-wise measures can provide more accurate results in contrast to pixel-wise methods, but the computation time increases since, for each pixel in an image, more pixels around are involved, which can limit usage on window-based approaches in rapid estimation algorithms.

A Census transformation was formulated in [31] as intensities-dependent non-parametric transform. The base Census estimation works as follows. For a pixel *p* with pixel coordinates (u,v) in an image *I*, its intensity value is compared with other pixels around the reference, coordinates of which are defined in a set *M*:

(2) $$\begin{array}{c} I_{C}\left(u,v\right)=\underset{[i,j]\in M}{⨂}\xi \left(I\left(u,v\right),\;I\left(u+i,v+j\right)\right), \end{array}$$

where ⊗ stands for bit-wise concatenation, and pixel relations are defined as:

(3) $$\begin{array}{c} \xi (v_{1},v_{2})=\left\{\begin{array}{cc} 0, & v_{1}⩽v_{2}\\ 1, & v_{1}>v_{2} \end{array}\right.. \end{array}$$

Such estimations can be done in a dense way, taking all pixels within the window into consideration, or in a sparse way by defining the coordinates of specific pixels to be involved in the Census image. Originally, this function is considered to be applied to the single-channel image; in our work, it is extended to be performed on RGB images, treating every channel separately.

To compare values of Census pixels in different views and to generate the matching cost, the Hamming distance is used. For two images in Census-transformed light field $L_{c}$ with coordinates $\left(\hat{s},\hat{t}\right)$ and (s,t) :

(4) $$\begin{array}{c} C_{c}\left(u,v,d\right)=HD\left(p_{c}\left(u,v,s,t,d\right),L_{c}\left(\hat{u},\hat{v},\hat{s},\hat{t}\right)\right), \end{array}$$

where HD() is the Hamming distance function: for two vectors, $x_{i}$ and $x_{j}$ ( $|x_{i}\left|=\right|x_{j}|=n$ , here and further |…| denotes cardinality), it can be determined as a sum of elements with different values:

(5) $$\begin{array}{c} HD\left(x_{i},x_{j}\right)=\sum_{k=1}^{n}x_{ik}⊕x_{jk}, \end{array}$$

where ⊕ stands for exclusive disjunction.

**Figure 4:**
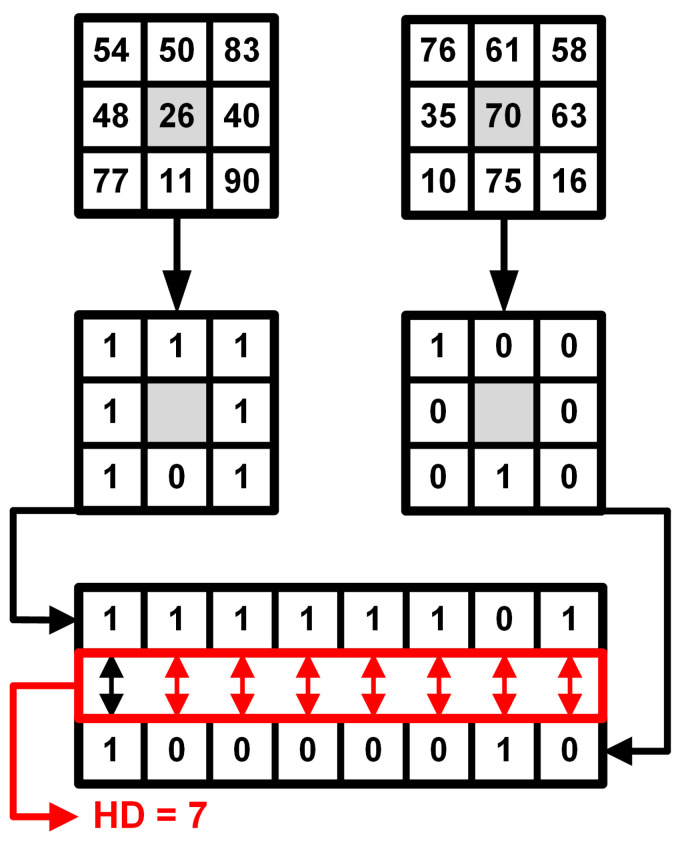
Visualization of image Census transformation and Hamming distance estimation.

Figure 4 demonstrates the principles of Census transformation and Hamming distance estimation. For every view in *V*, cost is generated by matching between this view and the opposite view on the same axis. In general, matching costs collected from two images might suffer from a big amount of ambiguities, which will negatively affect the estimation of disparity map by introducing noise to the image. The generated matching cost needs additional optimization to make it usable for accurate disparity estimation with a low noise level.

### 3.5. Semi-Global Matching

To solve this issue, we use a widely known Semi-Global Matching (SGM) method, proposed in [32]. This method can be considered as the optimal one between local-only matching cost collection and the global cost optimization, which can provide the most accurate results, but with significant computational load.

For each pixel p=(u,v) and d∈T , after traversing in direction *r*, formulated as a two-dimensional vector *r* = { Δu , Δv }, aggregated cost $L_{r}$ is

(6) $$\begin{array}{cc} \; & L_{r}\left(p,d\right)=C\left(p,d\right)+\\ \; & \min\;(L_{r}\left(p-r,d\right),\\ \; & L_{r}\left(p-r,d-1\right)+P1,\\ \; & L_{r}\left(p-r,d+1\right)+P1,\\ \; & \min_{t}\;L_{r}\left(p-r,t\right)+P2), \end{array}$$

where P1 and P2 are penalty parameters for neighborhood disparities, P2⩾P1 . Costs are then summarized among all directions:

(7) $$C_{S}\left(p,d\right)=\sum_{r}\;L_{r}\left(p,d\right).$$

### 3.6. Disparity Map Forming

From the matching cost, the disparity value for every pixel *p* can be estimated using the winner-takes-all (WTA) method as:

(8) $$D_{V_{i}}\left(p\right)=\underset{d}{\arg\min}\;C_{s}\left(p,d\right).$$

Despite the matching cost being subject to SGM, some noise pixels can still be present. For that, a median filter applied, where for every disparity map pixel, the median value of its neighborhood is associated.

### 3.7. Consistency Checks and Merging

After performing the previously described steps, we obtain a set of four disparity maps $D_{V}$ . Each of these maps is used for the consistency check. First, disparity maps are projected on a plane of reference view $\left(\hat{s},\hat{t}\right)$ . To do so, the order of views in Equation (1) is modified from $\left(\hat{s}-s\right)$ and $\left(\hat{t}-t\right)$ to $\left(s-\hat{s}\right)$ and $\left(t-\hat{t}\right)$ , where *s* and *t* are related to the actual view position in the light field, and for every pixel $d=D_{V_{i}}\left(u,v\right),i=1...\left|V\right|.$

Next, for each reprojected $D_{V_{i}}$ , we check the matching pixel in its opposite view $D_{V_{o}}$ (based on the original views placement) for their inequality:

(9) $$|D_{V_{i}}\left(u,v\right)-D_{V_{o}}\left(u,v\right)|<\varphi ,$$

where φ stands for confidence threshold. Every pixel of the fused initial disparity map $D_{C}$ is computed as the average of the corresponding pixel values from $D_{V}$ , for which the condition presented in Equation (9) is met. The pixel is discarded as uncertain if this condition is not true.

### 3.8. Per-Layer Disparity Filtering

Quality of the disparity image obtained with Hamming distance matching may contain noise elements for individual pixels, which would be difficult to remove with a median filter. For that, we propose a filter, based on per-layer decomposition of the initial disparity map. For every available disparity hypothesis d∈T , the new image, containing only pixels with disparity value *d*, is created:

(10) $$D_{C_{d}}\left(u,v\right)=\left\{\begin{array}{cc} D_{C}\left(u,v\right), & D_{C}\left(u,v\right)=d\\ 0, & D_{C}\left(u,v\right)\neq d \end{array}\right..$$

This image is a subject for the morphological closing operation [33], which stands for erosion, followed by dilation. Filtered disparity maps are combined back with preserving the presence of already associated pixels by processing the decomposed disparity maps from far to near. The resulting set of images is combined with the disparity map, used for the further borders generation step.

### 3.9. Holes Filling

After consistency check, merging and following filtering, the disparity map has a considerable amount of pixels without associated disparity value. In order to reduce the number of such pixels, we use a holes filling technique based on the neighborhood pixel information and color consistency. For a missing disparity pixel, the filling procedure is based on the median value of nearby values within a window of a certain size.

For the pixels, placed on the edges, such filling can lead to associating false values. To prevent it, we use values from a corresponding color light field view. Color pixels in the window are checked for their Euclidean distance from the reference pixel being below the threshold, based on which they are involved in the median value estimation.

**Figure 5:**
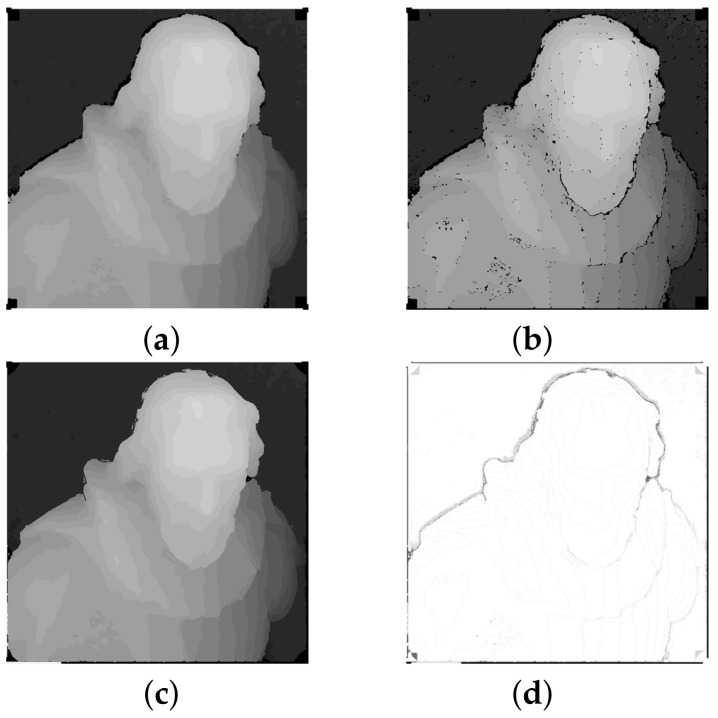
Initial disparity post-processing steps: (**a**) initial disparity map, (**b**) per-layer disparity filtering, (**c**) holes filling, (**d**) difference between (**a**,**c**).

This algorithm is performed iteratively, and the stopping criteria of this method are defined as the number of iterations and the number of non-empty pixels. To prevent jamming of the method on the same pixels after the third iteration, the window size is increased logarithmically and the threshold is relaxed accordingly. Figure 5 demonstrates the application of these steps to the initial disparity map.

## 4. Final Disparity Map Estimation

This section describes how the previously estimated coarse disparity map is used for computations involving more light field views.

### 4.1. Generation of Borders

The initial disparity map serves for creation of computational limitation for disparity hypothesis range. It fulfills two purposes. First, the generation of matching cost from many light field views is a time-consuming task. Limitation of the disparity search range by the bordering information reduces the running time of the matching procedure. Second, due to the ambiguities from the matching cost estimation, the wrong estimations and noise pixels can be present in the final disparity map. Bordering information prevents the appearance of these issues, which will be demonstrated later in Section 6.4.

$D_{C}$ is used for generation of boundaries for the further estimation. These boundaries will limit matching cost generation in the whole light field space. Two structures named high and low borders ( $D_{H}$ and $D_{L}$ respectively) are generated by using the border threshold λ in such a manner:

(11) $$\begin{array}{c} D_{H}\left(u,v\right)=D_{C}\left(u,v\right)+\lambda ;D_{L}\left(u,v\right)=D_{C}\left(u,v\right)-\lambda . \end{array}$$

The values, which lie outside of predefined disparity range $(D_{H}>d_{max}$ , $D_{L}<d_{min})$ are saturated accordingly. Invalid values from $D_{C}$ are marked in the corresponding borders for re-computation on the whole disparity range *T*.

### 4.2. Light Field Bordered Matching

The final disparity map can be estimated either from all images of the light field, or from the light field subset. Practically, it would be useful to utilize the views, lying on a cross from the reference. It can save the computational efforts on collecting the matching cost, whereas quality of the disparity would not be too much affected.

Matching cost for a pixel (u,v) for each possible disparity hypothesis *d*, lying in a range $\left[D_{L}\left(u,v\right),D_{H}\left(u,v\right)\right]$ is found as a sum of compared values in all cross-lying views:

(12) $$C_{S}\left(u,v,d\right)=\sum_{s=1}^{\left|s\right|}M(p\left(u,v,\hat{s},\hat{t},0\right),p\left(u,v,s,\hat{t},d\right))+\sum_{t=1}^{\left|t\right|}C(p\left(u,v,\hat{s},\hat{t},0\right),p\left(u,v,\hat{s},t,d\right)),$$

where |s| and |t| are the numbers of views in the spatial light field dimensions, and M() is a comparison function.

The selection of this function depends on the origin and quality of the data. For synthetic data, usage of Euclidean distance is a fair choice, as it is done in our evaluation:

(13) $$M\left(p_{1},p_{2}\right)=∥p_{1}-p_{2}∥.$$

However, for real-world scenarios, one would prefer Hamming distance-based comparison on Census-transformed images or more robust metrics like zero-normalized cross correlation [34].

Steps from Section 3.5 and Section 3.6 are applied to $C_{S}$ for obtaining final disparity map $D_{S}$ .

### 4.3. Sub-Pixel Refinement

Disparity map, computed with this method, contains only the values up to a pixel and can not be considered as accurate. As a post-processing step, we estimate the sub-pixel values of disparity pixels based on the matching cost. Usually, it is done by fitting a parabola to the neighboring cost values, associated with the disparity. However, this approach can produce a certain error, since, based on histogram analysis, the interpolated values are not equally distributed.

In this work, we use a technique called Symmetric-V interpolation, proposed by Haller et al. in [35].

For every pixel (u,v) , the values of interpolated image $D_{I}$ are computed as:

(14) $$\begin{array}{c} D_{I}\left(u,v\right)=D_{S}\left(u,v\right)+\\ \left\{\begin{array}{c} +\left(0.5-0.25\left(\frac{{\left(M3-M1\right)}^{2}}{{\left(M2-M1\right)}^{2}}+\frac{\left(M3-M1\right)}{\left(M2-M1\right)}\right)\right);M2>M3\\ -\left(0.5-0.25\left(\frac{{\left(M2-M1\right)}^{2}}{{\left(M3-M1\right)}^{2}}+\frac{\left(M2-M1\right)}{\left(M3-M1\right)}\right)\right);M2⩽M3 \end{array}\right.\\ M1=C_{S}\left(u,v,d\right),M2=C_{S}\left(u,v,d-1\right),M3=C_{S}\left(u,v,d+1\right) \end{array}$$

## 5. Point Cloud Processing

The disparity map, described in the previous section, is further used as initialization for the point cloud, which will be optimized using all light field images. Such representation allows processing of all parts of the scene, presented in light field images, but are not visible in the reference view of the light field.

### 5.1. Point Cloud Conversion

For the point cloud generation, the disparity map $D_{I}$ needs to be converted to the depth map, based on the common focal length of light field views *f* and the distance between two adjacent views on one axis *b*, which remains the same for all view pairs based on the light field parameterization:

(15) $$\begin{array}{c} D_{Z}\left(u,v\right)=\frac{fb}{D_{I}\left(u,v\right)}. \end{array}$$

Depth values are used for getting the 3D points *P* as:

(16) $$\begin{array}{c} P=\left[XYZ\right];Z=D_{Z}\left(u,v\right)\\ X=Z\frac{v}{f};Y=Z\frac{u}{f} \end{array}$$

### 5.2. Additional Views Analysis

To include the information for scene points, which are not visible in the reference light field view, we define the reference views in the corners of the light field. The initial disparity map $D_{C}$ , estimated in Section 3.6, is reprojected to the new reference view with the principles from Section 3.7. The reprojected image is then used for the generation of bordering information for the further estimation of final disparity map for the new reference view based on the cross-lying views, repeating steps from Section 4.

The point clouds are obtained by applying Equations (15) and (16) and transposed to the viewpoint of the original reference view. For multiple point cloud registration, we use a classical Iterative Closest Points (ICP) approach [36]. While ICP defines the needed transformation for all points, we remove the already preserved ones from the new point clouds by their projection to the original reference view plane and deleting the matching points. It allows for constructing the joined point cloud by just combining the point sets. For optimization purposes, the information about point origin is stored alongside with the point.

### 5.3. Nonlinear Optimization

Every point cloud is separately projected to various light field viewpoints. By that, we are trying to find value of *Z*, which minimizes the error between the projected and presented pixel in light field views:

(17) $$\begin{array}{c} \sum_{s=1}^{\left|s\right|}∥\hat{p}\left(u,v,s,\hat{t},d\right)-p\left(u,v,s,\hat{t},d\right)∥+\sum_{t=1}^{\left|t\right|}∥\hat{p}\left(u,v,\hat{s},t,d\right)-p\left(u,v,\hat{s},t,d\right)∥, \end{array}$$

where $\hat{p}$ is the projected pixel, estimated by the principles from Equation (16). Based on the point origin, it is optimized only on the frames, where the point is visible. For simplification, we define the possible configuration of viewpoints based on the cross-lying light field views.

Output on the nonlinear optimization step contains some specific noise, which requires additional post-processing efforts. For reducing such noise, we found that the Combined Bilateral Filter (CBF), proposed by Wasenmüller et al. in [37], suits best. It composes the classical bilateral filter with joint bilateral filter, published in [38].

## 6. Results

### 6.1. Synthetic Dataset

Evaluation of our method is done with a four-dimensional Light Field Benchmark [39] on a synthetic dataset by Honauer et al. [11]. Twelve synthetic scenes are used for the comparison; each of them is represented by the 9 × 9 light field, collected from 8-bit RGB images with 512 × 512 pixel resolution. The images from the dataset used in the benchmark are divided into three categories: "training" for parameter adjustment and evaluation, "stratified" with difficult cases and "test" for "blind" verification. Disparity and depth maps are provided for "training" and "stratified" categories.

**Figure 6:**
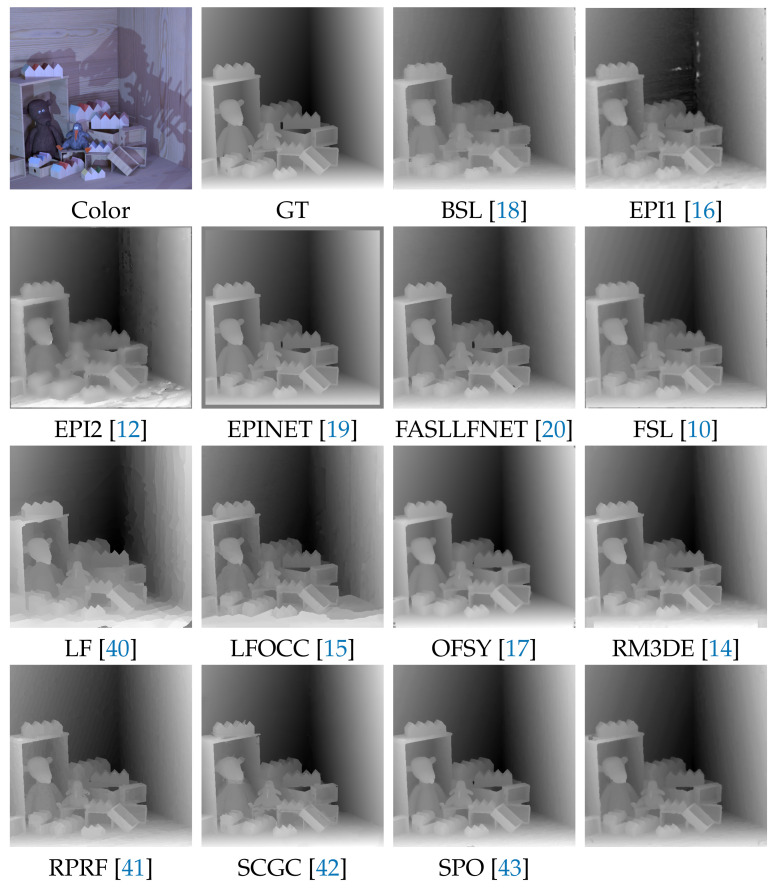
Qualitative results for "dino" scene from 4D Light Field Benchmark [11].

We provide a comparison of the proposed algorithm with the state-of-the-art methods, presented in Section 2: BSL [18], FASTLFNET [20], EPI1 [16], EPI2 [12], EPINET [19], FSL [10], LF [40], LFOCC [15], OFSY [17], RM3DE [14], RPRF [41], SCGC [42], and SPO [43]. Qualitative comparison for one of the light field images from "training" category is presented in Figure 6. Demonstration of the results on all scenes is presented on the web-site of 4D Light Field Benchmark [39].

### 6.2. Evaluation

The benchmark provides various metrics, on which algorithms can be evaluated. We provide results of the comparison on three metrics: the percentage of pixels where the absolute difference between the result and the ground truth is greater than the threshold, which is set to 7% ( BadPix ), mean square error over all pixels ( MSE ), and the maximum absolute disparity error of the best 25% of pixels ( Q25 ). The results of the evaluation on these metrics are presented in Table 1. The benchmark provides various photo-consistency metrics, which are not covered in this paper and can be found of the 4D Light Field Benchmark [39].

### 6.3. Algorithm Settings

Every scene shares all of the algorithm parameters except the disparity range, which is adjusted according to the scene configuration file. The values are set empirically based on the optimal values of the evaluation on three metrics from Section 6.2.

Census transformation from Section 3.4 uses a window of size 9 × 7. SGM penalties P1 and P2 are set to 30 and 150 for the initial disparity map estimation from Section 3. Due to the different comparison formula, these penalties in Section 4 are set to 20 and 40. For both scenarios, the number of traversing directions for SGM equals 4.

Confidence threshold φ for the consistency check and merging in Equation (9) is set to 2. Holes filling algorithm in Section 3.9 uses 25 iterations as the stopping criteria of the optimization. Initial window size is set to 5 and initial threshold for the distance between color values of the pixels is set to 5. Border threshold λ from Equation equals 1. CBF from Section 5.3 uses standard deviation values of 0.5 and 2.5. Window size for median-based filters is set to 3.

### 6.4. Discussion

**Figure 7:**
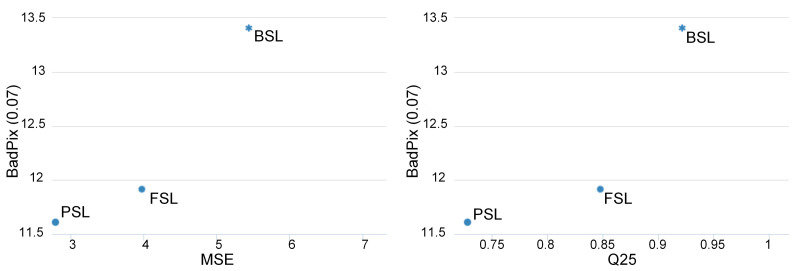
Comparison of the proposed algorithm (PSL) with its predecessors (BSL [18], FSL [10]) on the median of three metrics (lower is better), generated by [39].

Figure 7 shows how the result of the presented algorithm compares with its predecessors [10,18]. Overall, the quantitative results of our algorithm are consistent with the baseline. Values of three metrics for the benchmark were improved compared to the preceding algorithms, as demonstrated in Figure 7.

**Figure 8:**
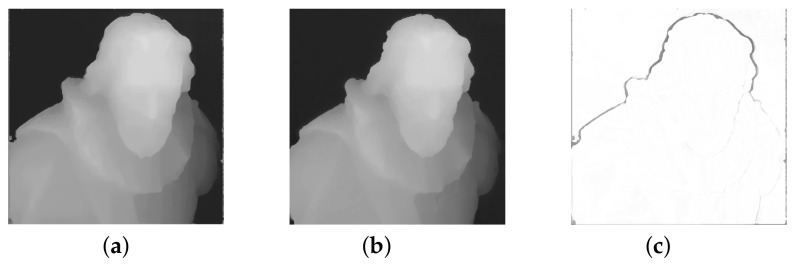
Effect of the per-layer disparity filtering and holes filling: (**a**) disparity map from FSL [10], (**b**) disparity map from proposed method, (**c**) difference between two disparity maps.

Figure 8 shows the difference between predecessor [10] and the proposed algorithm. Two main changes can be observed. First, the smoothness of surfaces in the proposed algorithms is improved, as can be seen in Figure 8a,b. It can be observed on both final and refined disparity maps. This happens partially due to a change in the subpixel refinement algorithm from the parabola fitting to Symmetric-V. However, most of the smoothness is brought by a point cloud refinement step. It is not limited to the discrete matching cost values, unlike the interpolation step. In total, this corrects the step effect on disparity values, which was strongly observable in [10].

Second, per-layer disparity filtering together with holes filling provides significant changes for the final result. It makes the reconstruction look sharper and closer to the color projection. It can be seen in Figure 8c that the proposed algorithm provides different reconstruction on edges, which usually was a spot of ambiguities for the matching algorithm. It happens because pixels around edges were considered as a part of a neighborhood disparity layer due to the nature of the matching algorithm, which considers the interpolated color values of pixels among light field views. However, some wrong pixels can still "survive" the filtering, as it can be seen on the left side of the statue’s head in Figure 8b. Potentially, it can be fixed by repeating the per-layer disparity filtering and holes filling several times.

Table 2 shows the quantitative difference of algorithm configurations with and without per-layer disparity filtering and holes filling, performed on a subset of images from the benchmark. It can be observed that filtering techniques not only affect the boundaries of the images, but also improve the accuracy of the algorithm.

Although the smoothness of surfaces is improved in our method, in terms of the benchmark metrics, our result is worse compared to deep learning methods. However, the advantage of our approach is that there is no need to provide training data. Such approach can be easily extended to the different configurations of cameras and to be used on various scenes as well, providing not perfect, but reasonable results.

Experiments with adaptive Census window did not show any significant improvement. In addition, in this approach, the dense window for Census transformation was used instead of a sparse one, unlike in our previous works. We found that the accuracy of initial disparity estimation is higher in such configuration.

**Figure 9:**
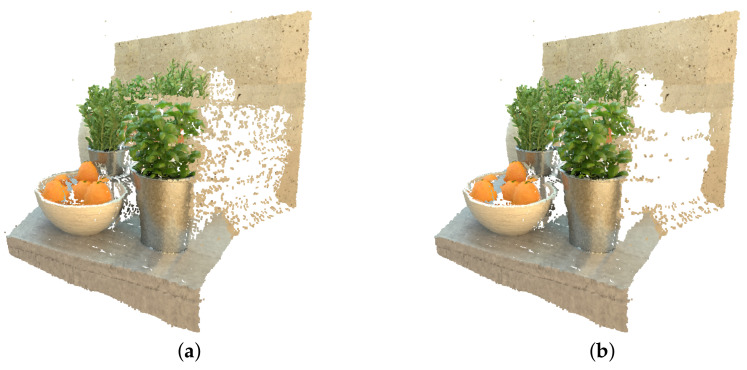
Effect of disparity boundaries on the point cloud: (**a**) point cloud without borders, (**b**) point cloud with borders.

Usage of borders significantly reduces the number of sampled hypotheses. Due to the change of domain from disparity maps to point cloud, a new advantage of using the boundaries was observed. Previously, only the running time-related changes were noted; however, it turns out that image initialization also prevents the creation of noise pixels on the areas of big disparity values transition, as it can be observed in Figure 9.

A nonlinear optimization step requires a good initialization. In addition, such optimization on a full disparity range can unfortunately create additional false estimations. For that, the searching range is limited to 1.5 pixels around the initial value from the final disparity map.

One way of improvement of this step, as well as general generation of bordering information, is related to utilization of matching cost confidence measurements. Different thresholds can be used based on the accuracy for the specific pixel. A modern overview of methods for that is presented in [44].

Unlike previous approaches, the running time of the proposed one is significantly higher. The main reason for that is the nonlinear optimization. We are currently investigating ways of reducing the running time for these operations.

The presented method was performed on a central processing unit (CPU). It limits the running time, since no parallelism was exploited. One way of making this method faster is by bringing its parts to graphics processing units (GPU). Since most of the operations are performed separately per pixel, it can be done in parallel. More complicated steps, such as SGM, can be paralleled by the traversing directions.

## 7. Conclusions

In this paper, we proposed an extension of the light field depth estimation method with the nonlinear point cloud refinement. Evaluation of our approach against state-of-the-art methods shows that results are comparable to the baseline result and improve its predecessors. Further work will try to reformulate the algorithm with principles of deep learning and to improve the running time by utilizing paralellism of GPUs and downscaled initial structures.

## Figures and Tables

**Table 1:** Evaluation of different algorithms with general metrics on 4D Light Field Benchmark [11].

	BadPix			MSE			Q25	
	Median	Average		Median	Average		Median	Average
BSL [18]	13.41	12.74		5.43	7.28		0.92	1.01
EPI1 [16]	22.89	24.32		3.93	5.98		1.00	1.23
EPI2 [12]	22.94	22.65		5.72	8.24		0.71	0.81
EPINET [19]	**3.38**	**4.93**		**1.21**	2.48		0.34	**0.34**
FASTLFNET [20]	8.24	9.07		1.61	**2.46**		0.57	0.58
FSL [10]	11.92	12.95		3.97	6.64		0.85	0.95
LF [40]	16.15	16.19		7.96	9.13		0.58	0.61
LFOCC [15]	18.45	17.58		2.8	6.69		1.70	1.60
OFSY [17]	11.33	12.04		5.43	7.03		**0.32**	0.37
RM3DE [14]	7.99	10.22		1.46	3.92		0.73	0.72
RPRF [41]	9.89	10.02		3.76	5.68		0.66	0.64
SCGC [42]	10.21	14.3		3.94	6.58		1.04	1.09
SPO [43]	8.78	8.47		3.31	3.97		0.60	0.71
PSL (proposed)	11.61	12.79		2.78	5.14		0.93	0.89

**Table 2:** Average results on "training" subset of 4DLFB [39] for the configuration with and without per-layer disparity filtering and holes filling.

	BadPix	MSE	Q25
With	9.89	3.57	0.74
Without	10.23	5.72	0.72

## Abbreviations

The following abbreviations are used in this manuscript:

CPU	Central Processing Unit
EPI	Epipolar–Plane Image
ICP	Iterative Closest Points
GPU	Graphic Processing Unit
SGM	Semi–Global Matching
